# Prognostic variables in patients with primary soft tissue sarcoma of the extremity and trunk treated with neoadjuvant radiotherapy or neoadjuvant sequential chemoradiotherapy

**DOI:** 10.1186/1748-717X-8-60

**Published:** 2013-03-14

**Authors:** Meena Bedi, David M King, Mikesh Shivakoti, Tao Wang, Eduardo V Zambrano, John Charlson, Donald Hackbarth, John Neilson, Robert Whitfield, Dian Wang

**Affiliations:** 1 Department of Radiation Oncology, Medical College of Wisconsin, 8701 Watertown Plank Rd, Milwaukee, WI, 53045, USA; 2 Department of Orthopaedic Surgery, Medical College of Wisconsin, Milwaukee, WI, USA; 3 Department of Biostatistics Center, Medical College of Wisconsin, Milwaukee, WI, USA; 4 Department of Pathology, Medical College of Wisconsin, Milwaukee, WI, USA; 5 Department of Medical Oncology, Medical College of Wisconsin, Milwaukee, WI, USA; 6 Department of Plastic Surgery, Medical College of Wisconsin, Milwaukee, WI, USA

**Keywords:** Sarcoma, Preoperative radiation therapy, Preoperative chemotherapy, Prognostic factors, Survival

## Abstract

**Background:**

Neoadjuvant radiotherapy (NRT) is an effective strategy to treat soft tissue sarcomas (STS). However, the role of neoadjuvant chemoradiotherapy (NCRT) remains to be determined.

**Methods:**

From May 1999 to July 2010, 112 patients with localized STS of the extremity and trunk who were treated with NRT or NCRT followed by surgery were retrospectively reviewed. Clinical outcomes including overall survival (OS), disease-free survival (DFS), and distant metastasis free survival (DMFS) were calculated using Kaplan-Meier survival analyses. Prognostic variables were determined by univariate (UVA) and multivariate analyses (MVA).

**Results:**

Median follow-up was 37 months. Median RT dose was 50 Gy. Forty-nine patients received NCRT. Overall limb-preservation rate was 99% and local control was 97%. The estimated 3-year OS, DFS, and DMFS were 86%, 68%, and 72%, respectively. Age was the only variable to predict for OS, DFS and DMFS on UVA. Age ≥ 70 predicted for poor OS, stage III disease predicted for poor DFS and DMFS, and the addition of chemotherapy predicted for improved DMFS on MVA.

**Conclusions:**

Excellent rates of local control and limb-preservation were observed in patients with primary STS treated with neoadjuvant therapy followed by surgery. Neoadjuvant sequential chemotherapy followed by radiotherapy may be considered for young patients with stage III STS.

## Introduction

The management of soft tissue sarcomas (STS) of extremity and trunk has evolved in the past two decades. Limb-salvage surgery combined with either pre-operative or post-operative radiotherapy has now become the standard of care over amputation [1-3]. Although there is no definitive evidence to support a survival benefit of neoadjuvant radiotherapy over adjuvant radiotherapy, results from a recent Phase III study demonstrate a significant advantage of neoadjuvant radiotherapy in the reduction of late radiation morbidities, such as fibrosis, joint stiffness and edema [4]. Reduction of late radiation morbidities is important in patient’s quality of life, as these morbidities are most likely irreversible. Additional advantages of neoadjuvant radiotherapy are a decrease in target volume, radiation dose and tumor seeding during sarcoma resection, as well as an occasional reduction in tumor mass that might facilitate complete resection [5-7].

Neoadjuvant chemoradiotherapy has been utilized in the management of high-risk STS of extremities [8-11]. For instance, neoadjuvant chemotherapy (MAID regimen) interdigitated with neoadjuvant radiotherapy (44 Gy in 22 fractions) has been shown to decrease the rate of distant metastasis (DM) and to increase disease free survival (DFS) and overall survival (OS) in patients with STS of the extremity and trunk with ≥ 8 cm tumors compared with historical control [8]. This prompted RTOG 9514, which was a single-arm phase II trial that enrolled 64 patients with intermediate-to-high grade, ≥ 8 cm STS of the extremity or torso with expected margin-negative (R0) resection. The 3-year loco-regional failure was 18% if amputation was considered a failure and 10% if not. Estimated 5-year rates of DFS, distant metastasis-free survival (DMFS), and OS were 56.1%, 64.1%, and 71.2%, respectively. However, significant toxicities associated with this aggressive neoadjuvant chemoradiotherapy regimen have precluded widespread use of this regimen [9].

Neoadjuvant sequential chemoradiotherapy has been known to be less toxic than neoadjuvant concurrent/interdigitated chemoradiotherapy and is commonly used to treat high-risk STS at many institutions. However, it remains to be determined whether this approach provides a survival benefit in patients with STS of the extremity and trunk. In this study, we analyzed a cohort of patients with primary STS of the extremity and trunk who received neoadjuvant radiotherapy with or without sequential neoadjuvant chemotherapy at our institution. The aim of this retrospective study was to determine the clinical and pathologic variables that predict for improved OS, DFS, and DMFS in patients with STS treated with this regimen.

## Materials and methods

This research was reviewed and approved by the Institutional Review Board (IRB) and all investigators completed training in both human research and patient privacy.

### Patients

All patients with primary STS of the upper and lower extremities and trunk who received radiation with or without chemotherapy followed by surgical resection between May 1999 and October 2010 were reviewed. Exclusion criteria included metastatic disease on initial presentation, age < 18 years old, STS of locations other than the extremity or trunk, recurrent sarcomas at first presentation, and histopathologic types demonstrating rhabdomyosarcoma, extraosseous primitive neuroectodermal tumor, Ewing sarcoma, osteosarcoma, Kaposi’s sarcoma, angiosarcoma, aggressive fibromatosis, or dermatofibrosarcoma protuberans. Patients who did not have complete medical records including treatment information and a pathology report, and follow-up of less than 6 months were also excluded. Patients were staged according to the 2009 American Joint Committee on Cancer (AJCC) system seventh edition.

### Statistical analysis

The sample size for this analysis was 112 patients. Potential prognostic variables assessed were location, grade**,** size, depth, age, percent necrosis on surgical specimens, whether neoadjuvant chemotherapy was administered, and the number of chemotherapy cycles. The 2, 3 and 5-year OS, DFS, and DMFS rates were estimated using the Kaplan-Meier estimate of the survival function. The log-rank test was used to compare two survival curves. Univariate and multivariate analyses were performed to determine prognostic variables in correlation with the above survivals. For multivariate analysis, the Cox proportional hazards model was used. All potential risk factors were first tested for affirmation of the potential hazards assumptions. No factors were found to violate the proportional hazards assumption. A step-wise model building procedure was then performed to develop multivariate models for each outcome. For all analyses, the type I error was maintained at 0.05 and all tests were two-sided. All statistical analyses were performed in SAS, ver. 9.1 (Cary, NC).

## Results

### Patient characteristics

There were 112 patients with stage I-III STS of extremity and trunk that were included in this analysis. Median age was 54.5 years old, with a range between 18 to 92 years. Patient characteristics are listed in Table 1.

**Table 1:** Patient characteristics

		**Frequency (%)**
**Tumor Stage**	I	17
	II	14
	III	69
**Histology**	Undifferentiated/MFH	35
	Liposarcoma	26
	Myxofibrosarcoma	17
	Leiomyosarcoma	10
	Synovial Cell Sarcoma	9
	Other	10
**Location**	Trunk	20
	Extremity	80
**Grade**	Low	17
	Intermediate	5
	High	78

The most common histology was malignant fibrous histiocytoma (30.3%). Other histologies included liposarcoma (23.2%), myxofibrosarcoma (14.3%), leiomyosarcoma (8.9%), synovial sarcoma (8.0%), extraskeletal myxoid chondosarcoma (2.7%), malignant peripheral nerve sheath tumor (3.6%), solitary fibrous tumor (3.6%), spindle cell sarcoma, NOS (1.8%), epitheliod sarcoma (1.8%), and fibrosarcoma (1.8%). Nineteen patients (17%) had low grade, 6 (5%) had intermediate grade, and 87 (78%) had high grade disease.

All patients underwent external beam radiotherapy followed by limb-sparing, wide-local excision. The median dose administered was 50 Gy in 25 fractions using either three-dimensional radiotherapy or intensity modulated radiation treatment. Of the patients with accessible radiation records, a three-dimensional technique was used in 79.4% and IMRT was used in 20.6% of patients. Surgical resection was performed 4–8 weeks after completion of radiation treatment. Negative margins, which are defined as tumor on ink, were achieved in 92% of all cases upon wide local excision. The limb-preservation rate was 99%. One patient underwent an amputation for a failed infected reconstruction with vascular flap. Immediate flap reconstructions were performed in 53 (47.5%) of patients.

Forty-nine patients (40%) received neoadjuvant chemotherapy prior to the initiation of radiation. All chemotherapy was doxorubicin and ifosfamide-based regimens (Table 2). Twenty-eight patients (57%) received 3 or more cycles of chemotherapy, 20 of these patients received less than 3 cycles of chemotherapy, and 63 (56%) patients received no chemotherapy. The number of cycles of chemotherapy was unknown in one patient. Neoadjuvant chemotherapy was often given to young patients (<70 years old) with clinical stage III (large, deep, intermediate-to-high grade) STS. Neoadjuvant chemotherapy was given to 4 out of 48 (8.3%) patients ≥70 years old compared to 27 out of 64 patients (42.2%) who were <70 years old and did not receive chemotherapy (p<0.0001).

**Table 2:** Demographics of patients receiving neoadjuvant chemotherapy

**Variable**		**NRT**	**NCRT**
**Age**	≤70	39 (45%)	48 (55%)
	>70	21 (84%)	4 (16%)
**Stage**	I	18 (9.5%)	1 (5%)
	II	10 (62.5%)	6 (37.5%)
	III	33 (43%)	44 (57%)
**Grade**	Low	18 (95%)	1 (5%)
	Intermediate	4 (67%)	2 (33%)
	High	38 (44%)	49 (56%)
**Size**	≤5 cm	3 (30%)	7 (70%)
	>5 cm	49 (48%)	53 (52%)

NRT: neoadjuvant radiotherapy. NCRT: neoadjuvant chemoradiotherapy.

Adjuvant chemotherapy was also delivered in 14 patients who received neoadjuvant chemotherapy. Twelve patients received a doxorubicin-ifosfamide based regimen, one patient received an epirubicin-ifosfamide based regimen, and one patient received adjuvant chemotherapy at an outside clinic, but the regimen was not available for review. One patient received five cycles of adjuvant chemotherapy, 5 patients received two cycles, 2 patients received three cycles, and the remaining 6 received an undetermined number of cycles.

### Pathologic outcomes

Percent necrosis was documented in 102 of the 112 patients in our database. The original pathology reports were centrally reviewed by our sarcoma pathologist (EZ), who was blind to clinical outcomes. Ten patients had 100% necrosis of their tumor after neoadjuvant treatment, 14 patients had ≥99% necrosis, 23 patients had ≥95% necrosis, 20 patients had 80-94% necrosis, 20 patients had 50-79% necrosis, and 39 patients had less than 50% necrosis of their tumor.

### Clinical outcomes

Median follow-up was 3.1 years. Eight patients were lost to follow-up. The local control rate for the entire study population was 97% at this follow-up. Two of the 3 patients that failed locally did so after the development of distant metastasis. The distant metastasis rate for the study group was 27.6%. The median time to death was 23.5 months. The median time to disease progression was 16.3 months. The median time to distant metastasis was 15.6 months.

**Figure 1:**
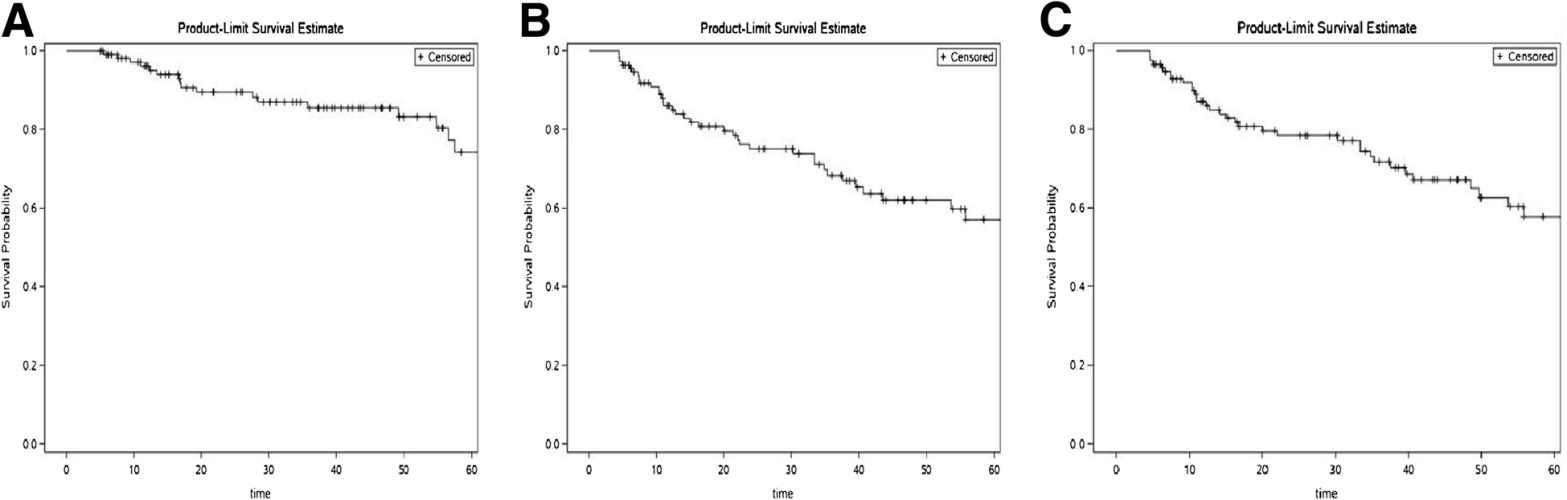
Overall survival (A), disease-free survival (B), distant metastasis free survival (C) of the entire group.

The median OS, DFS and DMFS was > 124 months (Figure 1A, 1B, and 1C), with a 3-year OS of 85.5%, 3-year DFS of 68.3%, and 3-year DMFS of 71.7%.

There was no significant difference in the OS (p=0.42), DFS (p=0.29), and DMFS (p=0.12) between T1 (tumor ≤5 cm) and T2 (tumor >5 cm) STS. There was no significant difference in OS (p=0.73), DFS (p=0.41), and DMFS (p=0.48) between deep and superficial sarcomas. There 3-year OS for low, intermediate and high grade disease was 93.7%, 83.3% and 84.6%, respectively (p=0.5). The 3-year DFS for low, intermediate and high grade disease was 83.1%, 76.2%, and 64.9%, respectively (p=0.2). The 3-year DMFS was 82.6%, 71% and 69%, respectively (p=0.2).

Necrosis assessed on pathology from the surgical specimen was grouped according to the degree of necrosis after the central pathology review. There was no significant difference in OS (p=0.4), DFS (p=0.37), and DMFS (p=0.71) in patients who had ≥95% vs. ≥80%-94% vs. ≥50%-79% vs. <50% necrosis.

**Figure 2:**
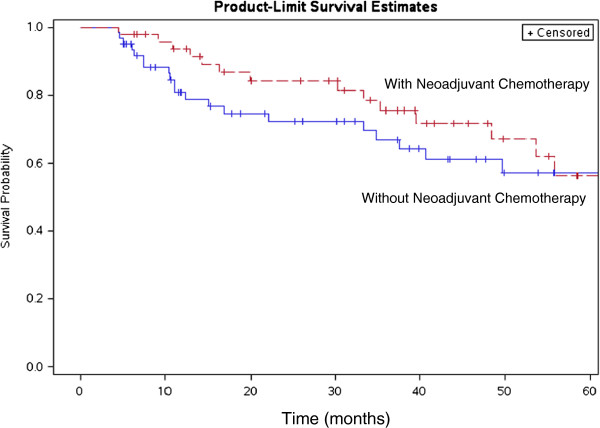
Distant metastasis free survival (DMFS) of patients with neoadjuvant chemotherapy versus without neoadjuvant chemotherapy.

On univariate analysis, age <70 predicted for OS (p=0.019), DFS (p=0.037), and DMFS (p=0.021). Stage also predicted for DMFS on UVA (p=0.017). No other variable was significant for OS, DFS or DMFS. On multivariate analysis, age ≥70 was significant for poor OS (p=0.026), stage III disease was significant for poor DFS (p=0.041) and DMFS (p=0.004), and the addition of neoadjuvant chemotherapy prior to preoperative radiotherapy was significant for improved DMFS (p=0.041) (Figure 2).

## Discussion

High local control rates have been demonstrated using multimodality therapy for STS. Yang et *al.,* showed that the addition of post-operative radiation to chemotherapy improved local control rates in both high and low grade tumors [12]. Several other studies have demonstrated high local control rates of approximately 90% at 5 years with preoperative radiotherapy with or without chemotherapy [4,13-16] (Table 3). Brant, et *al*., demonstrated a 91% local control rate in 58 patients with STS of the extremity or trunk who were treated with preoperative radiation alone followed by surgical resection. The University of Florida series was recently updated with an overall local control rate of 94%. Similarly, Barkley, et *al.* also had high rates of local control [13,14,17]. Studies that have compared preoperative radiation to post-operative radiation have also shown equivalent outcomes for local control [4,15,16]. Similarly, this study demonstrates a high local control rate of 97% at a median follow-up of 3.1 years for those treated with preoperative radiation therapy followed by limb-sparing surgery. This suggests that neoadjuvant radiotherapy combined with surgical resection does not compromise local control in STS of the extremity and trunk.

**Table 3:** Local control with neoadjuvant radiotherapy for primary soft tissue sarcomas of extremity and/or trunk

**Study**	**Institution**	**Number of pre-op patients & follow-up**	**Chemotherapy included**	**Median RT dose (Gy)**	**Local control**
Dagan , et *al.*	U of Florida	317	Yes	50.4	94%
		4.7 y			
O'Sullivan, et *al.*	NCI	93	No	50	93%
		3.3 y			
Sampath, et *al.*	Utah	293	Yes	50.4	93%
		5.25 y			
Zagars, et *al.*	MDACC	271	Yes	50	87%
		6 y			
Bedi, et *al.*	MCW	112	Yes	50	97%
		3.1 y			

Neoadjuvant chemotherapy is often preferred over adjuvant chemotherapy, although there is no level one evidence to support a survival advantage with the use of neoadjuvant chemotherapy in patients with STS. The Sarcoma Meta-analysis of STS patients enrolled into 14 randomized trials revealed that adjuvant chemotherapy was found to improve local recurrence-free survival by 6%, distant and overall recurrence-free survival by 10% [18]. However, proponents for neoadjuvant chemotherapy argue that oncologists are able to monitor response and alter or terminate therapy in patients who do not appear to be deriving any benefit. Gortzac, et. *al* reported results of a phase II study that examined patients with high-risk sarcomas who were randomized to surgery alone vs. three cycles of doxorubicin and ifosfamide prior to surgery. After a median follow-up of 7.3 years, the addition of chemotherapy failed to show any benefit in DFS or OS [19]. Authors concluded that this study did not have enough power to address survival. Therefore, neoadjuvant chemotherapy alone should not be routinely recommended to all patients with STS.

The results from this study show that the use of neoadjuvant sequential chemotherapy was associated with a higher DMFS in patients <70 years old with clinical stage III STS of the extremity and trunk. This is similar to the results of other studies compared with historical controls [8,9]. The mechanism of biological synergy from neoadjuvant sequential chemoradiotherapy remains unknown. Considering the rarity of STS and uncertain response of some STS to neoadjuvant chemotherapy, the authors would therefore recommend neoadjuvant sequential chemoradiotherapy be considered in treating patients <70 years old with stage III STS in centers with considerable multidisciplinary expertise and experience in managing these patients and where such an approach can be discussed for appropriate patients in the setting of a multidisciplinary sarcoma tumor board. As such, close monitoring of these patients are essential during the course of neoadjuvant chemotherapy. At our institution, patients <70 years old with stage III STS of the extremity and trunk are jointly evaluated by all disciplines.

The decision to administer neoadjuvant chemotherapy prior to radiation is dependent on several factors. Grade, tumor size, and histology all are taken into account, as well as patient factors such as performance status, cardiovascular and renal functioning and patient preference. Typically, chemotherapy is recommended in high grade STS with certain histologies that are ≥5cm in size or deep tumors. These histologies include synovial sarcoma, myxoid liposarcoma, dedifferentiated liposarcoma, poorly differentiated STS, and leiomyosarcoma. These patients are also offered neoadjuvant sequential adriamycin-ifosfamide chemotherapy for three cycles. These patients might receive immediate preoperative radiotherapy due to significant tumor pain or growth after 1 or 2 cycles of chemotherapy. All patients will have a CT scan of the chest and an MRI of the primary sarcoma after 2 cycles of chemotherapy. Those patients whose tumors are stable or shrink would proceed with the third cycle of neoadjuvant chemotherapy prior to preoperative radiotherapy. All of the above scenarios should be extensively discussed with these patients prior to initiating treatment.

Prognostic variables have been investigated in many retrospective studies [20-29]. Stevanovski, et *al.* have demonstrated that stage is prognostic in OS; other studies show that grade, size, and depth of tumor all are prognostic in terms of OS, disease-specific survival, and DMFS [19-25]. In this study, the UVA showed age ≥ 70 was the only variable that predicted for OS, DFS, and DMFS, and stage III disease predicted for poor DMFS. On MVA, age ≥70 was significant for poor OS, stage III disease was significant for poor DFS and DMFS, and the addition of neoadjuvant chemotherapy prior to preoperative radiotherapy was significant for improved DMFS.

Age at diagnosis has been demonstrated to be a prognostic indicator in several other studies [21,27-29]. Both Pisters, et *al.* and Gronchi et *al.*, showed decreased local control rates in patients with STS [21,27]. A study done by Collin et *al.*, revealed that survival was also impacted in patients who were > 53 years old at diagnosis, and Kaytan, et *al.* also showed poorer survival in patients > 50 years of age [28,29]. These studies corroborate our finding that advanced age at diagnosis may portend to poorer outcomes. Furthermore, a 12-year sarcoma-specific death nomogram has been created by Kattan and colleagues, which uses age as one of the nomogram predictor variables [30].

Treatment-induced tumor necrosis due to radiation with or without chemotherapy has been sparsely reported. Eliber, et *al*., showed improved 5-year survival rates of 80% vs. 62% in tumors with near-complete necrosis [11]. Similarly, MacDermed, et *al.* demonstrated a 5-year freedom-from-distant metastasis rate of 84.6% in patients who had a high rate of necrosis after treatment (≥90%) vs 19.9% in tumors that had less extensive necrosis [31]. However, a prospective phase II RTOG study on STS demonstrated that a near complete necrosis after neoadjuvant interdigitated chemoradiotherapy did not predict for any survival outcomes [8]. Similarly, results of this study showed the percent of necrosis after preoperative radiotherapy with or without chemotherapy did not impact OS, DFS, or DMFS. The reasons for the differences from our study are unclear and could be secondary to tissue sample selection that is not able to be examined in a retrospective study. There are also several issues about documenting percent of necrosis after neoadjuvant treatment for STS. First, there are no methods to differentiate between treatment-induced necrosis and tumor-related necrosis either through imaging studies or pathology examination. Second, the pre- and post-treatment percent of necrosis were not able to be compared in our study and other studies [11,31]. Third, percent of necrosis was not uniformly evaluated in above studies due to the large size of STS, as there was a technical challenge to evaluate entire specimen of large STS. Finally, different from many other types of human malignancy, high grade and large size are often associated with a significant increase in STS necrosis at initial diagnosis. This observation is confirmed in our study.

Limitations of this study include the inherent biases of a retrospective review and the relatively small sample size. Moreover, there were a heterogeneous group of tumors analyzed.

## Conclusions

Excellent rates of local control and limb-preservation were observed in patients with primary STS of the extremity and trunk treated with neoadjuvant radiation or neoadjuvant sequential chemoradiation followed by surgery. Age ≥70 years old was the only variable alone found to be prognostic for poor OS, DFS, and DMFS and stage III disease predicted for poor DMFS on UVA. Results of the MVA suggest that neoadjuvant sequential chemotherapy followed by radiation may be considered for young patients (<70 years old) with stage III STS of the extremity and trunk.

## Competing interest

The authors’ declare that they have no competing interest.

## Authors’ contributions

MB, DMK and DW were responsible for data collection, study concept and design, creating figures and tables, as well as manuscript preparation and revision. MS and TW were responsible for statistical analysis and figure creation with help from the statistical software. DMK, JC, DAH, JN, RW and DW staged each patient’s disease, performed surgical resections for pathology to review, treated patient’s according to recommendations and revised the manuscript. EAZ was responsible for pathological re-review of each case in this study and revisions of the manuscript. DW had the original data and was responsible supervision and confirming the final manuscript. All authors have read and approved the final manuscript.
